# Hypothalamic transcriptional expression of the kisspeptin system and sex steroid receptors differs among polycystic ovary syndrome rat models with different endocrine phenotypes

**DOI:** 10.6061/clinics/2017(08)09

**Published:** 2017-08

**Authors:** Rodrigo Rodrigues Marcondes, Kátia Cândido Carvalho, Gisele Giannocco, Daniele Coelho Duarte, Natália Garcia, José Maria Soares-Junior, Ismael Dale Cotrim Guerreiro da Silva, Manuel Maliqueo, Edmund Chada Baracat, Gustavo Arantes Rosa Maciel

**Affiliations:** I Laboratorio de Ginecologia Estrutural e Molecular (LIM 58), Disciplina de Ginecologia, Faculdade de Medicina FMUSP, Universidade de Sao Paulo, Sao Paulo, SP, BR; II Laboratorio de Endocrinologia Molecular e Translacional, Departamento de Medicina, Universidade Federal de Sao Paulo, Sao Paulo, SP, BR; III Laboratorio de Ginecologia Molecular e Proteomica, Departamento de Ginecologia, Escola Paulista de Medicina, Universidade Federal de Sao Paulo, Sao Paulo, SP, BR; IV Endocrinology and Metabolism Laboratory, Department of Medicine, West Division, University of Chile, Santiago, Chile

**Keywords:** Polycystic Ovary Syndrome, Hypothalamus, Animal Models, Kisspeptin, Testosterone

## Abstract

**OBJECTIVES:**

Polycystic ovary syndrome is a heterogeneous endocrine disorder that affects reproductive-age women. The mechanisms underlying the endocrine heterogeneity and neuroendocrinology of polycystic ovary syndrome are still unclear. In this study, we investigated the expression of the kisspeptin system and gonadotropin-releasing hormone pulse regulators in the hypothalamus as well as factors related to luteinizing hormone secretion in the pituitary of polycystic ovary syndrome rat models induced by testosterone or estradiol.

**METHODS:**

A single injection of testosterone propionate (1.25 mg) (n=10) or estradiol benzoate (0.5 mg) (n=10) was administered to female rats at 2 days of age to induce experimental polycystic ovary syndrome. Controls were injected with a vehicle (n=10). Animals were euthanized at 90-94 days of age, and the hypothalamus and pituitary gland were used for gene expression analysis.

**RESULTS:**

Rats exposed to testosterone exhibited increased transcriptional expression of the androgen receptor and estrogen receptor-β and reduced expression of kisspeptin in the hypothalamus. However, rats exposed to estradiol did not show any significant changes in hormone levels relative to controls but exhibited hypothalamic downregulation of kisspeptin, tachykinin 3 and estrogen receptor-α genes and upregulation of the gene that encodes the kisspeptin receptor.

**CONCLUSIONS:**

Testosterone- and estradiol-exposed rats with different endocrine phenotypes showed differential transcriptional expression of members of the kisspeptin system and sex steroid receptors in the hypothalamus. These differences might account for the different endocrine phenotypes found in testosterone- and estradiol-induced polycystic ovary syndrome rats.

## INTRODUCTION

Polycystic ovary syndrome (PCOS) is a highly prevalent heterogeneous condition mainly characterized by hyperandrogenism, chronic anovulation and polycystic ovaries 1. Another abnormality that is common in women with PCOS is aberrant gonadotropin-releasing hormone (GnRH) secretion, which favors higher production of luteinizing hormone (LH) and an increase in androgen production by the ovaries 2. However, the precise mechanisms underlying LH hypersecretion in PCOS are not well known.

The kisspeptin system, which includes kisspeptin, dynorphin A and neurokinin B/tachykinin 3, is essential for GnRH/LH pulse control, and it is believed that impairments in this system might contribute to endocrine dysfunction in PCOS 3. Furthermore, evidence suggests that neuroendocrine dysfunction in insulin signaling alters GnRH/LH secretion and impair reproductive cyclicity 4.

Exposure to either estrogen or androgens in neonatal life can induce chronic anovulation and polycystic ovaries resembling human PCOS during rat adulthood 5. However, different exposures may result in various endocrine phenotypes 5. For instance, animals exposed to androgens during the neonatal period present increased levels of LH and testosterone. This phenotype is not observed in the estrogen model 6. The aim of this study was to investigate transcriptional changes in the kisspeptin system and GnRH pulse regulators in the hypothalamus as well as factors related to LH secretion in the pituitary in PCOS rat models induced by testosterone or estradiol.

## MATERIALS AND METHODS

### Animals

The experimental procedures were described previously 6. In brief, thirty female Wistar rats at 2 days of age were allocated into the following groups for the induction of experimental PCOS: subcutaneous injection of 1.25 mg of testosterone (TG; n=10) 7 or 0.5 mg of estradiol (EG; n=10) 8. Control animals received a single injection of olive oil (vehicle) (CG; n=10). At 90-94 days of age, under anesthesia, blood was obtained from the abdominal aortic artery, animals were decapitated, and the hypothalamus and pituitary gland were removed and stored in RNAlater solution (Ambion; Thermo Fisher Scientific, USA) for 24 h and frozen until use. The measurement of serum levels of LH, FSH and testosterone is described in our previous study 6. This study was approved by the Institutional Ethics Committee of Faculdade de Medicina da Universidade de São Paulo under protocol number 151/10.

### Quantitative real-time polymerase chain reaction (qRT-PCR)

RNA extraction, cDNA synthesis and qRT-PCR conditions were performed as described elsewhere 9. Taqman® assays manufactured by Life Technologies were selected to analyze the gene expression of *Gnrh*, *Gnrhr*, *Kiss1*, *Kiss1r*, *Tac3*, *Tacr3*, *Esr1*, *Esr2*, *Ar*, *Cyp19a1*, *Pdyn* and *Oprk1* in the hypothalamus and *Gnrhr*, *Kiss1*, *Kiss1r*, *Insr* and *Ar* in the pituitary (Table 1). The data were analyzed using Sequence Detection Software (version 2.0.6), and Ct values were transformed to quantities using the comparative Ct method (ΔΔCt).

### Statistical analysis

Statistical analyses were performed with GraphPad Prism (version 6.05; GraphPad Software, USA). ANOVA followed by the Bonferroni *post hoc* test was performed to analyze data with a normal distribution. For skewed data, the Kruskal-Wallis test followed by Dunn’s *post hoc* test was performed. A value of *p*<0.05 was considered to indicate statistical significance. F-ratios (F) are described for significant differences. The degrees of freedom was equal to 29 for all analyses.

## RESULTS

**Figure 1:**
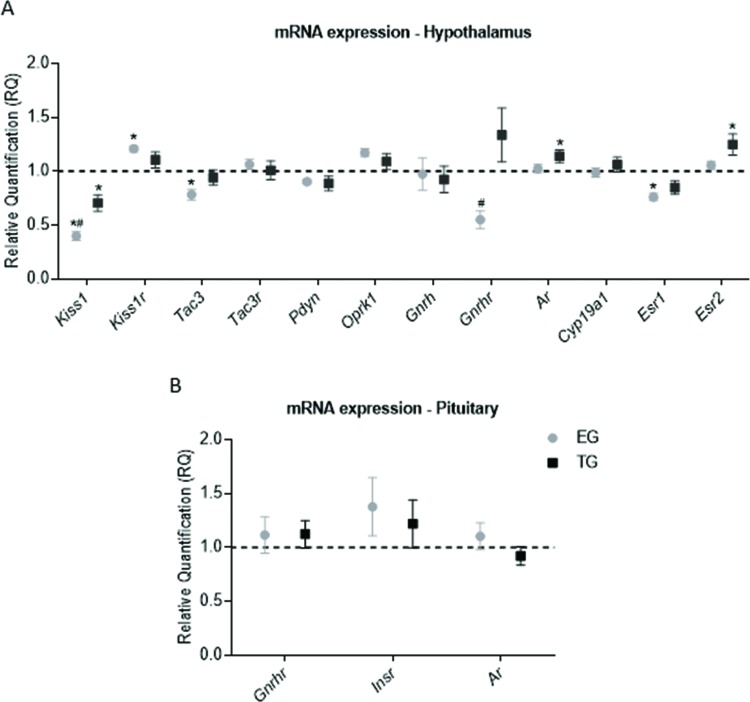
Transcriptional expression in the hypothalamus and pituitary. The data are shown as the means ± SEM. ANOVA followed by Bonferroni’s test was performed for data with a normal distribution, and the Kruskal-Wallis test followed by Dunn’s post hoc test was performed for skewed data. (A) mRNA expression in the hypothalamus. (B) mRNA expression in the pituitary. * - vs the CG (*p*<0.05), # - EG vs TG (*p*<0.05).

Treated rats had closed vaginas, and all rats in the CG had normal estrous cycles. In the hypothalamus, kisspeptin (*Kiss1*), neurokinin B (*Tac3*), and estrogen receptor (ER)-α (*Esr1*) were downregulated in the EG relative to the CG (*p*=0.0001, F=35.92; *p*=0.01, F=5.193; and *p*=0.0015, F=8.342, respectively). The expression levels of *Kiss1* and GnRH receptor (*Gnrhr*) were lower in the EG than in the TG (*Kiss1*: *p*=0.01, F=35.92; *Gnrhr*: *p*=0.01, F=6.748), and the expression of the gene encoding the KISS1 receptor (*Kiss1r*) was higher in the EG than in the CG (*p*=0.007, F=5.142). *Kiss1* was also expressed at a lower level in the TG than in the CG (*p*=0.001, F=35.92). TG rats exhibited upregulation of the androgen receptor (*Ar*) and ER-β (*Esr2*) genes compared to CG animals (*p*=0.04, F=3.522; and *P*=0.02, F=4.497, respectively). The expression of *Gnrh*, *Oprk1*, *Pdyn*, *Tacr3* and *Cyp19a1* did not differ significantly among the groups (Figure 1A). No significant difference was observed in pituitary gene expression (Figure 1B), and *Kiss1* and *Kiss1r* mRNA levels were undetectable.

## DISCUSSION

Our group showed in a previous study that testosterone or estradiol exposure in neonatal life leads to different PCOS-like phenotypes in adult female rats. Testosterone-induced PCOS rat models exhibit increased LH and testosterone levels, anovulation, and polycystic ovaries, while estradiol-induced PCOS rat models exhibit anovulation and polycystic ovaries but no alterations in the serum levels of gonadotropins or testosterone 6. In the current study, we aimed to further understand the heterogeneity of PCOS endocrine phenotypes by studying the hypothalamic neuroendocrine transcriptional profile of the kisspeptin system and GnRH pulse regulators as well as factors related to LH secretion in the pituitary of these two PCOS rat models.

It has been hypothesized that androgenized rat models have increased GnRH expression 10, and this event, in part, seems to be mediated by the interaction of androgens with androgen receptors (ARs) in the hypothalamus 11. Feng et al. 10 have shown that the AR is co-expressed in GnRH-neurons of adult female rats. In rats exposed to androgens in this study, an upregulation of *Ar* occurred in the hypothalamus. *In vitro*, AR activation has been shown to increase GnRH-neuron firing activity and GnRH secretion 12, 13.

Kisspeptin is an important factor involved in the stimulation of GnRH/LH secretion as well as the GnRH/LH surge that induces ovulation 3. Androgen and estrogen exposure downregulate kisspeptin gene expression, with this effect being more pronounced in estrogen-exposed rats. The downregulation of kisspeptin might be related to anovulation, because those animals seem to be unable to generate the GnRH/LH surge 6. In contrast, the KISS1 receptor gene was upregulated in rats exposed to estrogen, which is possibly a compensatory mechanism for low levels of kisspeptin.

Despite downregulation of the kisspeptin gene, androgen-exposed rats presented increased levels of LH. However, GnRH pulsatility, which stimulates gonadotropin secretion in a GnRH pulse-dependent manner, as well as LH secretion, is modulated by many factors 14. Other factors might be involved in LH hypersecretion in androgen-exposed rats.

Another factor that might contribute to anovulation in estrogen-exposed rats is the lower level of ER-α mRNA because female rats treated with an ER-α antagonist could not achieve a GnRH/LH surge, even with induction of the LH surge by intracerebroventricular administration of kisspeptin. However, the central administration of an ER-β antagonist did not impair the GnRH/LH surge in female rats 15. Mice with ER-β knockdown, but not those with ER-α knockdown, can generate positive estrogen-mediated feedback for the LH surge, and the action of a specific ligand of ER-α induces the LH surge 16. ER-α is expressed at a higher level in kisspeptin neurons than in GnRH neurons, while ER-β is the main estrogen receptor in GnRH neurons 17, which suggests that estrogen-exposed rats cannot achieve positive kisspeptin neuron-mediated estrogen feedback for GnRH/LH secretion. Androgen-induced PCOS rats exhibited upregulation of the ER-β gene. The role of ER-β in reproduction is not completely known, but this receptor also interacts with an androgen metabolite called 3β-diol 18. However, the role of this metabolite in the GnRH or kisspeptin system is still unknown.

Neurokinin B is considered an important factor in the modulation of GnRH secretion because loss-of-function mutations in the genes encoding both neurokinin B and its receptor induce hypogonadotropic hypogonadism 19. Furthermore, the central administration of a neurokinin B receptor agonist increases LH secretion in female rats 20. Downregulation of the neurokinin B gene (*Tac3*) may be related to the state of anovulation in estradiol-exposed rats and the lower levels of LH in the estradiol-exposed rats relative to the androgen-exposed rats. Additionally, testosterone- and estradiol-induced PCOS rat models differ regarding GnRH receptor mRNA expression in the hypothalamus, which might contribute to the neuroendocrine differences in those rat models.

To the best of our knowledge, this is the first study to compare the transcriptional expression in neuroendocrine organs in PCOS rat models with different endocrine phenotypes. However, this is a preliminary study, and one limitation is the absence of protein expression analysis.

**Figure 2:**
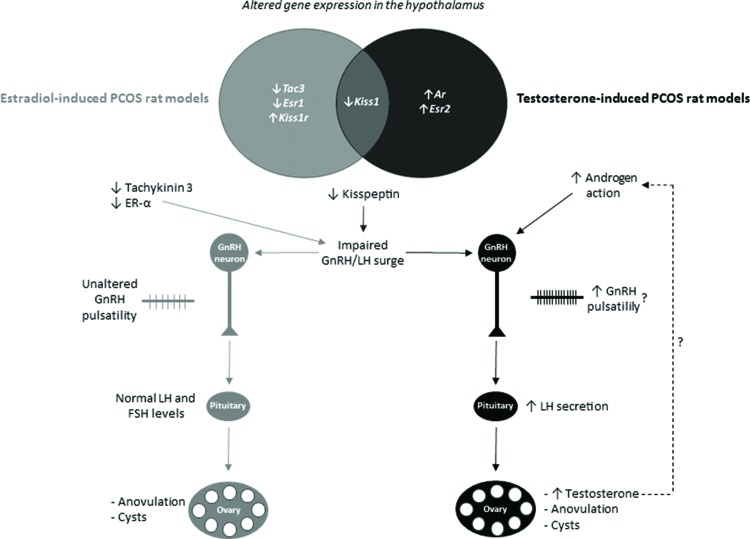
Differential transcriptional expression in the hypothalamus and hypothesized effects on the endocrine phenotypes of estradiol- or testosterone-induced PCOS rat models. The diagram shows genes with altered expression in the hypothalamus of estradiol- or testosterone-induced PCOS rat models; these two models only share the downregulation of the kisspeptin gene (*Kiss1*). Downregulation of the kisspeptin gene might contribute to anovulation in both testosterone- and estradiol-induced PCOS rat models because kisspeptin is essential for the gonadotropin-releasing (GnRH)/luteinizing hormone (LH) surge that precedes ovulation. The testosterone-induced PCOS rat model seems to have increased androgen-mediated stimulation of GnRH neurons, which might increase GnRH pulsatility and increase LH secretion by the pituitary. Increased LH secretion per se stimulates the ovaries to increase testosterone production. In the estradiol-induced PCOS rat model, downregulation of the estrogen receptor-α (ER-α) and tachykinin 3 genes seems to impair the GnRH/LH surge and ovulation. ↓, decreased; ↑, increased.

In summary, we found that testosterone- and estradiol-induced PCOS rats with different endocrine phenotypes exhibit differential transcriptional expression of members of the kisspeptin system and sex steroids in the hypothalamus. It is possible that different insults during development activate various neuronal circuitries, which might be related to the heterogeneity of PCOS. The hypothesized effects of altered hypothalamic transcriptional expression on endocrine phenotypes are summarized in Figure 2. These differences seem to be caused by various mechanisms and might account for the different neuroendocrine and endocrine phenotypes found in androgen- and estrogen-induced PCOS rats and women with PCOS.

## AUTHOR CONTRIBUTIONS

Maciel GA and Marcondes RR conceived and designed the study. Marcondes RR, Carvalho KC, Giannocco G, Duarte DC and Garcia N performed the experiments. Marcondes RR, Maciel GA, Baracat EC, Maliqueo M, Soares-Junior JM, Silva ID and Carvalho KC analyzed and interpreted the data. Marcondes RR and Maciel GA wrote the manuscript. All authors read and approved the final version of the manuscript.

## Figures and Tables

**Table 1:** Description of the probes and primers used in the study.

Gene symbol	Gene name	TaqMan ID	RefSeq
Reference genes			
*Actb*	Actin, Beta	4352340E	NM_031144.3
*Gapdh*	Glyceraldehyde-3-Phosphate Dehydrogenase	4352338E	NM_017008.4
*Ppia*	Peptidylprolyl Isomerase A (Cyclophilin A)	Rn00690933_m1	NM_017101.1
Target genes			
*Gnrh1*	Gonadotropin-Releasing Hormone 1	Rn00562754_m1	NM_012767.2
*Gnrhr*	Gonadotropin-Releasing Hormone Receptor	Rn00578981_m1	NM_031038.3
*Kiss1*	Kisspeptin	Rn00710914_m1	NM_181692.1
*Kiss1r*	KISS1 Receptor	Rn00576940_m1	NM_023992.2 and NM_001301151.1
*Tac3*	Tachykinin 3	Rn00569758_m1	NM_019162.2
*Tacr3*	Tachykinin Receptor 3	Rn00566955_m1	NM_017053.1
*Pdyn*	Prodynorphin	Rn00571351_m1	NM_019374.3
*Oprk1*	Opioid Receptor, Kappa 1	Rn01448892_m1	NM_017167.2
*Esr1*	Estrogen Receptor 1 (ER Alpha)	Rn01640372_m1	NM_012689.1
*Esr2*	Estrogen Receptor 2 (ER Beta)	Rn00562610_m1	NM_012754.1
*Ar*	Androgen Receptor	Rn00560747_m1	NM_012502.1
*Insr*	Insulin Receptor	Rn00690703_m1	NM_017071.2
*Cyp19a1*	Cytochrome P450, Family 19, Subfamily A, Polypeptide 1	Rn00567222_m1	NM_017085.2
